# Climate Change and Mental Health Nexus in National Climate Policy—Gaps and Challenges

**DOI:** 10.5334/aogh.4718

**Published:** 2025-04-04

**Authors:** Lea Schlatter, Manasi Kumar, Pushpam Kumar

**Affiliations:** 1 Swiss Federal Institute of Technology (ETH Zurich), Zurich, Switzerland; 2 PhD Professor, Institute for Excellence in Health Equity and Department of Population Health, New York University School of Medicine, 180 Madison Avenue, NY 10016 USA; 3 United Nations Environment Programme, Nairobi, Kenya

**Keywords:** climate change, mental health, nationally determined contributions, global health policy

## Abstract

*Background:* Climate change is increasingly recognized as a driver of mental health disorders, exacerbating conditions such as anxiety, depression, and post-traumatic stress. However, climate policies rarely address mental health considerations.

*Objective:* This study investigates the extent to which mental health is incorporated into national climate adaptation policies, specifically Nationally Determined Contributions (NDCs), from countries classified as high or very high risk according to the INFORM index.

*Methods:* We conducted a systematic literature review and policy analysis of NDCs from 38 high-risk countries. A keyword-based approach was used to assess the frequency and depth of mental health references in climate policies.

*Findings:* Only 8 of 38 countries explicitly referenced mental health in their NDCs. Most policies prioritized physical health, with little attention given to the psychological impacts of climate-related disasters. Vulnerable populations, including children, women, and individuals with preexisting mental health conditions, remain largely unaddressed in these national policies.

*Conclusions:* There is a significant gap in the integration of mental health impact and interventional indicators within climate change policies. Greater investment in interdisciplinary research and policy reforms are needed to ensure climate adaptation strategies address both physical and mental health concerns.

## 1. Introduction

Climate change, characterized by extended alterations in temperature and atmospheric conditions, stands as one of the most pressing issues of the 21st century, driven primarily by human activities such as the combustion of fossil fuels resulting in greenhouse gas emissions, reduction in forest coverage and green spaces, and increased population pressure stressing natural resources. Specifically, the combustion of fossil fuels results in the emission of greenhouse gases (GHGs), which trap heat from the sun and consequently raise temperatures. According to the Intergovernmental Panel on Climate Change (IPCC), global surface temperatures have risen significantly, with an increase of 1.09 °C in 2011–2020 compared with 1850–1900, before the Industrial Revolution. Moreover, average surface temperatures have increased more rapidly since 1970 than in any other 50-year period over at least the last 2000 years [1]. Consequently, alterations in temperature have cascading effects on various weather phenomena, such as drought, wildfire, and rising sea levels [2].

The repercussions of climate change are not evenly distributed, often disproportionately affecting those who bear the least responsibility [3]. For instance, climate change was responsible for 400,000 global deaths in 2010, with 98% of these fatalities occurring in the Global South [4]. Recognizing the urgency of this issue, numerous agreements have been pursued to address it. Among them is the Paris Agreement, which aims to limit global warming to below 2 °C, with each party setting and achieving its goals to reduce greenhouse gas emissions and enhance climate resilience. An important outcome of this agreement is the Nationally Determined Contributions (NDCs), in which 196 countries have committed to reducing their emissions and adapting to climate impacts [5].

Both climate change and mental health are gaining prominence in discussions surrounding public policy and global initiatives, yet mental health remains inadequately addressed and insufficiently understood [6]. According to the World Health Organization (WHO), mental health is defined as Mental health is a state of mental well-being that enables people to cope with the stresses of life, realize their abilities, learn well and work well, and contribute to their community. One of WHO’s key messages is that “there is no health without mental health” [7], emphasizing the need to incorporate mental health within overall health and now increasingly championed within development programming. Mental ill-health alters both human health and societal welfare, such that Kumar *et al*. estimated that the societal expenses associated with mental disorders resulting from shifts in climate-related risks, air pollution, and insufficient access to green spaces are projected to reach nearly US$47 billion globally and annually by 2030. Moreover, in comparison with a baseline scenario where these environmental factors are maintained at 2020 levels, these costs are expected to escalate, reaching US$537 billion in 2050 [8].

While the impact of climate change has mainly focused on physical health, recent research efforts have begun to explore the nexus between climate change and mental health. This growing body of work underscores the various adverse effects that climate change can impose on mental well-being, including emotional distress, anxiety, depression, grief, and even an elevated risk of suicide ideation. For instance, Liu and colleagues demonstrated in a meta-analysis that each 1 °C temperature rise was linked to a 2.2% rise in mortality related to mental health and a 0.9% increase in morbidity [9]. Additionally, short-term exposure to increased temperature is associated with a higher risk of suicide- or mental-disorder-related mortality (RR = 1.024 per 1 °C increase), suicidal behavior (RR = 1.012 per 1 °C increase), and increased hospital access due to mental disorders (RR = 1.009 per 1 °C increase) [10]. This reinforces the urgent need to integrate mental health considerations into climate adaptation policies.

This evidence synthesis and policy review aims to explore the relationship between climate change and mental health, extending to scrutinize the NDCs, particularly focusing on targeted UN nation-states highly susceptible to climate change. NDCs are crucial as they represent a country’s voluntary commitments to climate action, outlining its plans for mitigation and adaptation. Scrutinizing these contributions allows for an assessment of the strategies and measures countries are willing to undertake to address mental health concerns in the context of climate change. Quantifying mental health, especially in relation to climate change, is challenging due to its multifaceted nature and numerous influencing factors [11]. Nevertheless, research on this topic is essential to grasp its mechanics and provide actionable insights for policymakers to formulate more comprehensive and inclusive climate policies.

## 2. Methods and Materials

### 2.1 Literature review and evidence analysis

In the first phase, we conducted a rapid literature review and evidence synthesis to investigate the potential connection between climate change and mental well-being. Our systematic screening of literature began in September 2023, using Google Scholar with the keywords ‘climate change’ and ‘mental health.’ The scope included various climate events (e.g., tropical cyclones, heatwaves, droughts, wildfires, hurricanes, floods) and mental health issues or disorders (e.g., depression, post-traumatic stress disorder (PTSD), suicide ideation, anxiety, schizophrenia). We restricted our search to English-language studies published from January 2016 onward to ensure contemporary relevance. Gray literature, such as reports and conference proceedings, was also excluded to ensure the rigor of peer-reviewed research. Ultimately, we selected 50 studies that met these criteria, including 32 review papers. Vulnerable groups identified in the literature are discussed in Section 3.1. Vulnerabilities to climate change can be defined by a combination of socioeconomic, environmental, and health-related factors. Vulnerable populations include individuals with preexisting mental health conditions, those living in poverty, Indigenous groups, and children, among others [12].

### 2.2 Policy analysis

In the second phase, we conducted a policy analysis focusing on countries (*N* = 39) identified as at very high and high risk for climate change impacts. Vulnerability measurement frameworks typically use composite indices that incorporate factors such as economic stability, healthcare access, and climate exposure risk. The Index for Risk Management (INFORM) assesses risk on the basis of hazard exposure, lack of coping capacity, and sociopolitical vulnerabilities, providing a multidimensional perspective on susceptibility to climate-induced mental health impacts. INFORM, a collaborative effort of UN agencies, donors, nongovernmental organizations (NGOs), and research institutions, aims to provide a unified global assessment of humanitarian risk. INFORM was selected due to its comprehensive and updated coverage of 191 countries, incorporating 50 indicators grouped into three dimensions: hazard and exposure, vulnerability, and lack of coping capacity. This index is calculated as follows [13]:

$$Risk\;=\;{\left(Hazard\;\&\;Exposure\right)}^{1/3}\times \;{\left(Vulnerability\right)}^{1/3}\times \;{\left(Lack\;of\;coping\;capacity\right)}^{1/3}$$

- *Hazard & exposure* gauge the probability of physical exposure to climate-related hazards.
- *Vulnerability* evaluates the predispositions of exposed populations to hazard-related impacts, considering economic, political, and social characteristics.
- *Lack of coping capacity* assesses a country’s disaster management capabilities, encompassing both institutional aspects (mitigation and preparedness) and infrastructure (emergency response and recovery).

According to INFORM, countries are categorized into five risk classes on the basis of a 0–10 scale: very low, low, medium, high, and very high risk. In the paper, we focused on countries categorized as high or very high risk, ensuring that our analysis targeted those most susceptible to climate-induced risks, especially mental health challenges.

- *Very High Risk:* This category includes 15 countries with risk scores between approximately 8.5 and 8.7.
- *High Risk:* A total of 24 countries fall under this category, with risk scores ranging from around 6.0 to 8.4.

Then, we examined the Nationally Determined Contributions (NDCs) to determine whether health issues were mentioned in national climate change policies. Our focus on NDCs from countries classified as very high and high risk according to the INFORM index provided significant depth to this research. To identify relevant content, we applied a systematic keyword search approach, using terms such as vulnerable, mental health, well-being, health, women, farmers, Indigenous, youth, children, and elderly, translated into the language of the document, for instance, French, Spanish, and Arabic, as needed. The NDCs were sourced from the United Nations Framework Convention on Climate Change (UNFCCC) NDC registry. When health topics were identified, we examined the frequency of mentions, the specific context in which mental health was addressed, and whether these mentions were accompanied by concrete actions, commitments, or impact measures, rather than being merely referenced. Additionally, a qualitative review was conducted to determine whether mental health was framed as a priority issue with dedicated strategies or an incidental mention within broader health discussions.

## 3. Results

### 3.1 Vulnerable groups

Climate change acts as an amplifier of existing issues, disproportionately affecting individuals who are less equipped to cope, such as children, the elderly, women, and those with preexisting mental health conditions [10]. Vulnerability can also stem from specific weather events such as extreme heatwaves, cyclones, house damage, and drought, each impacting different groups in unique ways.

For example, children and the elderly are prone to conditions such as post-traumatic stress disorder (PTSD) following climate-related disasters, while women face heightened risks of gender-based violence (GBV) and depression due to climate-induced migration. Individuals with preexisting mental health conditions are also more vulnerable, facing increased risks of PTSD and psychological distress [12, 14]. Additionally, lower-income communities, specific occupational groups, and minorities often bear the brunt of climate-related events, experiencing property loss, economic hardships, and social dislocation, all of which can exacerbate mental health challenges [15]. For instance, the decline in access to land among Innuits due to climate change has been linked to alarming rates of youth suicide, underscoring the profound impact of environmental shifts on cultural identity and mental well-being [16].

### 3.2 National climate change policy

**Figure 1:**
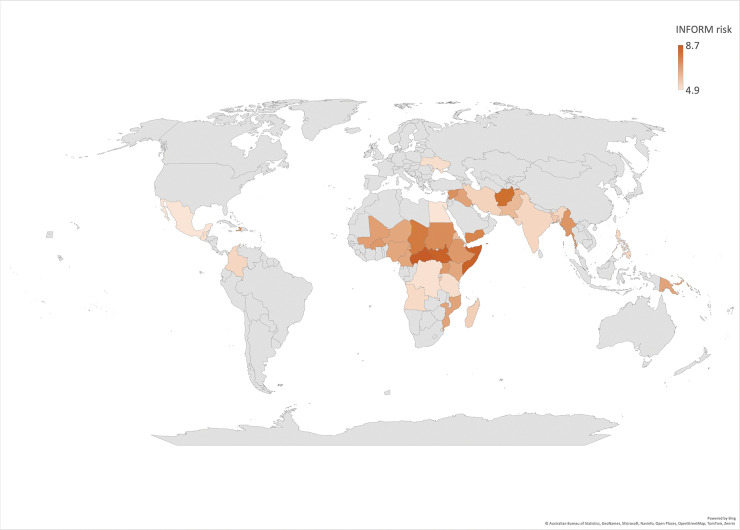
Global distribution of countries most at risk from climate change according to INFORM (2024). INFORM risk is measured on a scale from 1 to 10, with countries shaded by risk level. Scores above 6 indicate high risk, and scores above 8.4 indicate very high risk.

The previous section highlighted groups that are more vulnerable to climate change. Similarly, certain countries are disproportionately affected. Assessing which countries are most impacted requires robust metrics, such as those provided by INFORM. According to INFORM, the most vulnerable countries include, among others, the Central African Republic, Somalia, South Sudan, and Afghanistan. The detailed list of the 15 very high and 24 high risk countries is provided in the appendix. Notably, these countries also fall within global poverty hotspots and in the Global South as shown in Figure 1.

Since the health impacts of climate change are widely recognized, it is also worth examining whether mental health is given similar attention, and if so, to what extent compared with physical health. To assess whether these at-risk countries address the mental health impacts of climate change, we analyze their national climate policies, focusing on the integration of mental health considerations within their Nationally Determined Contributions (NDCs). The NDCs represent each country’s efforts to reduce national emissions and adapt to the impacts of climate change. The Paris Agreement mandates that each party prepare, communicate, and maintain successive climate plans they intend to achieve, pursuing national mitigation measures to meet their contributions. In the cases of Yemen and Madagascar, the Intended Nationally Determined Contribution (INDC) was considered. Iran, having not signed the Paris Agreement, does not have any NDCs.

#### 3.2.1 Physical health risks indicated in policies of countries at risk

Out of the 38 countries outlined in the initial list (excluding Iran, which does not have an NDC), 30 countries (60% of the very high-risk countries and 87.5% of the high-risk countries) have incorporated physical health considerations into their adaptive measures as part of their NDCs.

These health-related goals encompass strategies such as enhancing forecasting and preparedness for health disasters, increasing the number and quality of health facilities, and reducing the incidence of vector-borne diseases such as malaria and cholera or other climate change-related illnesses. For example, Eritrea has been implementing diverse initiatives, such as distributing mosquito nets, conducting public awareness campaigns, and utilizing mass media to inform the public about the impact of climate change on public health [17].

A consistent theme in these policies is the commitment to advancing education and research on health impacts associated with climate change, highlighting the goal of strengthening the knowledge foundation in this area. To enhance educational efforts, interventions need to expand beyond the current scope, addressing not only climate-related health impacts, but also conditions contributing to unrest and mental health challenges arising from climate change and environmental degradation. Countries such as the Central African Republic, Somalia, Sudan, Ethiopia, and Uganda emphasize improving water and hygiene quality to safeguard public health [18–22].

Some countries tackle health in their NDCs indirectly. For instance, Yemen and Afghanistan focus on enhancing disaster risk management, including flood and drought management, and raising awareness [23, 24]. Syria aims to develop early warning systems [25], while Haiti promotes the legal framework and affordable, appropriate insurance programs [26].

#### 3.2.2 Mental health policy status of countries at high risk

Now that physical health risks have been examined, it is worth seeing how mental health compares. Only 8 out of 38 countries (excluding Iran), representing less than 25%, have included actions to address mental health within their NDCs: Mexico, Republic of the Congo, Eritrea, Angola, Mozambique, Papua New Guinea, South Sudan, and Myanmar. Among the very-high-risk countries, Myanmar and South Sudan are the only ones mentioning mental health [27, 28]. South Sudan is committed to advancing human health and well-being through research funding, which could help identify vulnerable groups and formulate strategies to address mental health challenges. Similarly, Myanmar seeks to fortify its resilience against climate change-induced loss and damage by strengthening social protection, promoting gender considerations, and enhancing risk finance capacity. These proactive measures improve preparedness and recovery and play a pivotal role in enhancing the overall quality of life and well-being of Myanmar’s population.

Among the high-risk countries, Mozambique and Papua New Guinea note that a lack of water security negatively impacts the well-being of their populations [29, 30]. Additionally, Mexico highlights that biodiversity and environmental health directly affect the well-being of its population as part of its cultural heritage [31].

It is important to note that none of these countries specifically use the term mental health and that well-being was only briefly mentioned.

#### 3.2.3 Other considerations for thinking through additional vulnerabilities

Often, these most at-risk countries consider vulnerable communities in their climate policies. Some mention them briefly, while others have precise action plans. For instance, South Sudan has prioritized the empowerment of women by launching programs to improve the quality of cooking stoves [28]. Similarly, Somalia aims to strengthen the adaptive capacity of children, the elderly, women, and internally displaced people through social safety nets [19]. Additionally, Afghanistan and Sudan aspire to improve access to water and food for rural communities and farmers, along with enhancing their working conditions [20, 24].

Chad recognizes the vulnerability of women to climate change and has implemented programs for facilitated land access and social nets [32]. The Democratic Republic of Congo considers small farmers, children, and women as vulnerable groups, particularly in the agriculture sector. It also aims to integrate more women, youth, and Indigenous people into the decision-making processes of climate change policies [33]. Burkina Faso and Ethiopia focus on improving agricultural working conditions, specifically for women and children [21, 34]. Additionally, Tanzania advocates for accessible strategies to safeguard smallholder farmers and fishers from climate-related disruptions, including the provision of crop insurance [35].

## 4. Discussion

The findings of this study underscore the critical gap in integrating mental health considerations within national climate policies. While there is growing recognition of the physical health impacts of climate change, the psychological and emotional toll remains underexplored in policy discussions.

Our policy review further highlights discrepancies, at first, in how health issues are prioritized. While 30 of the 38 high-risk countries have incorporated physical health considerations into their climate policies, only 8 have acknowledged mental health, and even these references are often vague or indirect. This stark imbalance indicates that while governments recognize the need for climate-resilient healthcare infrastructure, they may not fully recognize all crucial elements that go into building a resilient health service model, especially as they fail to extend the same urgency to mental health needs during and in the aftermath of such climate-associated stressors and necessary services to address population distress. Additionally, the emphasis on immediate disaster response often neglects the long-term psychological consequences of climate change, such as the sequelae of post-traumatic stress disorder (PTSD), depression, substance-use, climate-related grief, and widespread eco-anxiety reported in young populations.

This oversight highlights a missed opportunity to develop holistic and ethical climate adaptation and mitigation strategies. Indeed, while climate-induced extreme weather events such as droughts, floods, and heatwaves are known to exacerbate stress, anxiety, and depression, Nationally Determined Contributions (NDCs) do not sufficiently capture these concerns, and national investment priorities often fail to allocate the necessary resources to mitigate such adverse health impacts. The vulnerability of marginalized groups—including children, the elderly, women, and individuals with preexisting mental health conditions—further exacerbates this issue. Without targeted interventions, these populations face disproportionate risks.

Addressing these gaps requires a multifaceted approach and efforts both globally and nationally. First, there is an urgent need to integrate mental health into climate resilience strategies, ensuring that NDCs explicitly include mental well-being alongside physical health. Governments should invest in community-based psychological treatment and mental health promotion programs, particularly for high-risk populations. Nature-based and nature-embedded mental health solutions could be an innovation to drive within NDCs given how much the populace relies on natural goods and services. Second, increased funding for interdisciplinary research is crucial to establishing stronger empirical links between climate stressors, mental health outcomes, and systems and services needed to empower and prepare populations. This evidence base can inform targeted policies that prioritize psychological resilience in climate adaptation plans. The INFORM risk classification can be used to strategically allocate resources and prioritize efforts.

## 5. Conclusions

Through the review, we have identified significant gaps in the inclusion of mental health in national climate policies compared with physical health. Despite growing evidence of the profound mental health impacts caused by climate change, only a small fraction of high-risk countries addresses this issue in their Nationally Determined Contributions (NDCs). This limited recognition suggests that mental health remains an overlooked aspect of climate adaptation strategies, leaving vulnerable populations exposed to risks that could be mitigated through more comprehensive policies.

To address this gap, policymakers must integrate mental health considerations into climate adaptation measures, ensuring they are given equal importance as physical health. Increased investment in research is essential to better understand the connection between climate change and mental well-being, which can guide the development of targeted interventions. A more holistic approach is needed to build resilience and protect mental health in the face of climate change. In the case of low- and middle-income countries, first, an agenda of shared problems across various ministries should be developed; second, an inter-ministerial body combining health and climate besides planning and fiscal should be formed to look into this; third, the inter-ministerial body must provide an authoritative assessment with clear and credible numbers on the mental health benefits of climate change action; fourth, the quantified and monetized benefits must be integrated in the planning and investment mechanisms through fiscal and monetary instruments, and finally, a community of practitioners should be developed through continued and concerted capacity development activities in public health, mental health, public policies, and climate change to estimate, monitor, and validate the findings on a regular basis across countries.

Such efforts would also contribute to bridging the existing inequity between the Global South, which bears most of the brunt of climate change impacts, and the Global North, which is comparatively less affected. Incorporating mental health into national climate strategies can help narrow this divide by providing at-risk countries with the tools and support necessary to strengthen their adaptive capacity and better safeguard their populations.

## Additional File

The additional file for this article can be found as follows:

Supplementary Table S1. Most at-risk countries (N = 39) according to their INFORM risk point (0 = low risk, 10 = high risk), the INFORM classification (very low, low, medium, high, very high) and their region.
