# Supplementary material for: Climate Change and Mental Health Nexus in National Climate Policy—Gaps and Challenges
Source: Ann Glob Health. 2025 Apr 4;91(1):19. doi: 10.5334/aogh.4718 (PMC11987854; doi:10.5334/aogh.4718)
Supplement: Supplementary Table S1. — Most at-risk countries (N = 39) according to their INFORM risk point (0 = low risk, 10 = high risk), the INFORM classification (very low, low, medium, high, very high) and their region. [file agh-91-1-4718-s1.pdf]

Supplementary Table

INFORM Risk	INFORM classification of risk	Countries	Region
8.7	Very high	Central African Republic	Central Africa
8.5	Very high	Somalia	East Africa
8.5	Very high	South Sudan	East Africa
8.1	Very high	Afghanistan	Southwest Asia
7.8	Very high	Chad	Central Africa
7.7	Very high	Democratic Republic of Congo (Congo DR)	Central Africa
7.5	Very high	Yemen	Middle East
7.3	Very high	Sudan	East Africa
7.2	Very high	Haiti	Central America
7.2	Very high	Syria	Middle East
7.1	Very high	Myanmar	Southeast Asia
7.0	Very high	Burkina Faso	West Africa
7.0	Very high	Ethiopia	East Africa
7.0	Very high	Uganda	East Africa
6.8	Very high	Mali	West Africa
6.7	High	Iraq	Middle East
6.7	High	Mozambique	East Africa
6.7	High	Papua New Guinea	Oceania
6.6	High	Cameroon	Central Africa
6.6	High	Kenya	East Africa
6.6	High	Niger	West Africa

6.6	High	Nigeria	West Africa
6.1	High	Pakistan	South Asia
6.0	High	Eritrea	East Africa
5.7	High	Bangladesh	South Asia
5.6	High	Burundi	East Africa
5.5	High	Madagascar	East Africa
5.4	High	Iran	Middle East
5.3	High	Colombia	Central America
5.3	High	India	South Asia
5.3	High	Philippines	Southeast Asia
5.2	High	Angola	Central Africa
5.1	High	Guatemala	Central America
5.1	High	Tanzania	East Africa
5.1	High	Ukraine	East Europe
5.0	High	Congo	Central Africa
4.9	High	Djibouti	East Africa
4.9	High	Egypt	Middle East
4.9	High	Mexico	Central America

Table S1: Most at-risk countries (N=39) according to their INFORM risk point (0 = low risk, 10 = high risk), the INFORM classification (very low, low, medium, high, very high) and their region.
